# Common definition for categories of clinical research: a prerequisite for a survey on regulatory requirements by the European Clinical Research Infrastructures Network (ECRIN)

**DOI:** 10.1186/1745-6215-10-95

**Published:** 2009-10-16

**Authors:** Christine Kubiak, Fernando de Andres-Trelles, Wolfgang Kuchinke, Karl-Heinz Huemer, Steffen Thirstrup, Kate Whitfield, Christian Libersa, Béatrice Barraud, Xina Grählert, Gabriele Dreier, Ruth Grychtol, Zsuzsa Temesvari, Gyorgy Blasko, Gabriella Kardos, Timothy O'Brien, Margaret Cooney, Siobhan Gaynor, Arrigo Schieppati, Nuria Sanz, Raquel Hernandez, Charlotte Asker-Hagelberg, Hanna Johansson, Sue Bourne, Jane Byrne, Adeeba Asghar, Jean-Marc Husson, Christian Gluud, Jacques Demotes-Mainard

**Affiliations:** 1 Institut Thématique Santé Publique, Institut National de la Santé et de la Recherche Médicale (Inserm), Paris, France; 2 KKS-Duesseldorf (KKSD), Heinrich-Heine University, Duesseldorf, Germany; 3 Medical University of Vienna (ATCRIN), Vienna, Austria; 4 Copenhagen Trial Unit (CTU), Centre for Clinical Intervention Research and the Danish Clinical Research Infrastructures Network (DCRIN), Rigshospitalet, Copenhagen University Hospital, Copenhagen, Denmark; 5 Ministry of Health Social and Family Affairs, Hungarian ECRIN Committee, Medical Research Council (HECRIN), Budapest, Hungary; 6 Molecular Medicine Ireland (ICRIN), Dublin; 7 National University of Ireland, Galway, Ireland; 8 Istituto di Ricerche Farmacologiche Mario Negri (IRFMN), Milano, Italy; 9 Hospital Clinic i Provincial de Barcelona (SCReN), Barcelona, Spain; 10 UK Clinical Research Network, Leeds, UK; 11 Karolinska Trial Alliance, Stockholm, Sweden; 12 ZKS - Clinical Trials Centre, University Medical Centre Freiburg, Freiburg, Germany; 13 Clinical Investigation Centre CH&U-INSERM 9301, Lille, France; 14 Departemento de Farmacologia (Medicina), Universidad Complutense, Madrid, Spain; 15 European Forum for Good Clinical Practice, Brussels, Belgium; 16 UK Clinical Research Collaboration, London, UK; 17 KKS, Medizinische Fakultät Carl Gustav Carus, Dresden, Germany; 18 Education and Research Centre, Wythenshawe Hospital, Manchester, UK

## Abstract

**Background:**

Thorough knowledge of the regulatory requirements is a challenging prerequisite for conducting multinational clinical studies in Europe given their complexity and heterogeneity in regulation and perception across the EU member states.

**Methods:**

In order to summarise the current situation in relation to the wide spectrum of clinical research, the European Clinical Research Infrastructures Network (ECRIN) developed a multinational survey in ten European countries. However a lack of common classification framework for major categories of clinical research was identified, and therefore reaching an agreement on a common classification was the initial step in the development of the survey.

**Results:**

The ECRIN transnational working group on regulation, composed of experts in the field of clinical research from ten European countries, defined seven major categories of clinical research that seem relevant from both the regulatory and the scientific points of view, and correspond to congruent definitions in all countries: clinical trials on medicinal products; clinical trials on medical devices; other therapeutic trials (including surgery trials, transplantation trials, transfusion trials, trials with cell therapy, etc.); diagnostic studies; clinical research on nutrition; other interventional clinical research (including trials in complementary and alternative medicine, trials with collection of blood or tissue samples, physiology studies, etc.); and epidemiology studies. Our classification was essential to develop a survey focused on protocol submission to ethics committees and competent authorities, procedures for amendments, requirements for sponsor and insurance, and adverse event reporting following five main phases: drafting, consensus, data collection, validation, and finalising.

**Conclusion:**

The list of clinical research categories as used for the survey could serve as a contribution to the, much needed, task of harmonisation and simplification of the regulatory requirements for clinical research in Europe.

## Background

Clinical research plays a vital part in making progress towards better knowledge, understanding of human health and disease and the development of new, safe and effective treatments. In Europe, at the national and at the European levels, there is a huge amount of pertinent legislation and guidance to clinical research and a thorough knowledge of the regulatory requirement is a prerequisite for supporting and conducting multinational clinical research. At the EU level, five key Directives are related to clinical research [1-5] and aim to provide a common legislative framework. However, the EU member states implemented the Directives into national laws with varying interpretation, thereby prevented the intended harmonisation of regulatory requirements. Therefore, it is not surprising that the conduct of international academic clinical research is highly complex and may hinder academic sponsors without support of a large team or a supportive structure as it is the case for big pharmaceutical companies or clinical research organisations (CROs) [6]. The academic research community in Europe needs an infrastructure to support their international clinical trials.

Supported by grants from the Sixth and Seventh Framework Programmes (FP6 and FP7), the European Clinical Research Infrastructures Network (ECRIN) was created to provide support to multinational clinical studies in Europe for the benefit of patients [7]. ECRIN is based on the connection of national networks of academic clinical research centres and clinical trials units from Austria, Denmark, Finland, France, Germany, Hungary, Ireland, Italy, Spain, and Sweden, Switzerland, and the United Kingdom. Each country has both one senior representative who is member of the governing body of ECRIN representing his national network and one European correspondent (trained in clinical research) working at the national coordination on the implementation of the ECRIN project.

During the FP6- funded project (TWG), different transnational working groups including at least one expert from each country participating in ECRIN, were established for analysing the context of clinical research in European countries and for preparing procedures and guidance documents to support the set-up and management of multinational studies. One of these groups was in charge of collecting the status of national regulations covering the whole spectrum of clinical research and the corresponding operating procedures. In order to achieve this objective, the group planned to design and conduct an international survey. As no commonly accepted definitions of clinical research categories existed in Europe, the first step in designing this survey was to delineate the relevant categories of clinical research, as interpreted by the ten regulation experts of the working group or currently defined by national laws.

Such a common categorisation could help investigators to deal with the current lack of harmonisation in a systematic way and, hopefully, be also of use to regulators and legislators in achieving harmonisation. The European Commission plans to perform by 2010 an assessment of the application of Directive 2001/20/EC, which specifically regulates the clinical trials with investigational medicinal products, with a view to make, if appropriate, legislative proposals. Therefore, the timing of the ECRIN survey which has a wider scope not limited to clinical trials with medicinal products only appears appropriate.

This article describes the development of the survey and the difficulties faced when trying to reach an agreement on the categories of clinical research, but the detailed results of the survey will be presented and discussed in further publications.

## Methods

### Definition of categories of research and development of the survey

The ECRIN working group on regulatory requirements and interaction with competent authorities was composed of two chairpersons and at least two representatives from each ECRIN national network (Austria, Denmark, France, Germany, Hungary, Ireland, Italy, Spain, Sweden, and United Kingdom): one expert in the field of regulations and regulatory requirements and the European correspondent.

The objective of the group was to collect information on current legislation and on the regulatory framework in the ten European countries previously listed. During the development of the survey the group experienced difficulties in reaching common understanding on the different major categories of clinical research. The group therefore embarked on this task as part of the development of the survey, in order to achieve common categories, having unanimous definitions and meanings.

The working tool used to achieve this objective was a standardised survey and its development underwent five main phases: drafting, consensus, data collection, validation and finalising.

#### Drafting phase

The objective of the survey was to analyse the regulatory situation and practices and provide the investigators and sponsors with a comprehensive status of regulatory requirements covering the whole spectrum of clinical research helping them to perform multinational studies.

The first step was to find common categories of research as basis for discussing the regulatory requirements to which the survey questions can be applied. The first proposal of categories was based on the similarity of key aspects in definition and meaning in national requirements.

#### Consensus phase

In order to ensure that the final version considered all national specificities and expressed an agreed common definition for clinical research categories, the members of the working group were contacted and invited to provide feedback on the proposed research categories and questions in the survey by e-mail and during teleconferences

#### Data collection phase

The survey was sent by email to the experts. The non-respondents were contacted again by email, then if necessary by phone in order to obtain all missing data. All the people contacted responded the survey.

In order to provide comprehensive responses, for each category and subcategory of clinical research, detailed questions were asked regarding regulatory requirements. In addition, questions with free text answers were included in order to allow the responders to give further details.

The completed survey was analysed by the leaders of the working group on regulatory requirements and interaction with competent authorities. When needed, the experts were interviewed by phone to give further information and explanation relating to the answers given on the survey. The preliminary results were discussed within the working group during several teleconferences and in face-to-face ECRIN meetings in Paris (19 and 20 May 2007) and in Brussels (19 and 20 May 2008).

Based on the final discussions the definition of categories were fine tuned and adapted to include national specificities.

#### Validation and finalising phase

The categorisation as well as the results of the survey were finalised by an informal validation step of representatives from the national competent authorities of the involved countries.

## Results and discussion

### Categories

The entire spectrum of clinical research was divided into seven major categories of clinical research to which the survey questions were applied [table 1]. The categories of clinical research defined by the working group are: the clinical trials on medicinal products (the only category already regulated as such at European level); clinical trials on medical devices; other therapeutic trials (including the group of surgery trials, transplantation trials, transfusion trials, trials with cell therapy, etc.); diagnostic studies; clinical research on nutrition; other interventional clinical research (including trials in complementary and alternative medicine, trials with collection of blood or tissue samples, physiology studies, etc.); and epidemiology studies.

**Table 1:** Seven major categories of clinical research

**Categories**	**Includes**
**Clinical trials on medicinal products**	-phase I to IV trials
	-biotherapy trials (gene therapy, tissue engineering and cell therapy)
	-biopharmaceutical trials (blood-derived products, monoclonal antibodies, recombinant proteins)
	-vaccines trials
	-fixed combination of medicinal products
	-multimodal trials

**Clinical trials on medical devices**	-devices alone
	-devices combined with medicinal products
	Devices are considered either as authorised (bearing the European conformity (CE) label and used within its indication or intended purpose), or as non-authorised (non CE labelled or used in another indication)

**Other therapeutic trials**	-radiotherapy trials
	-surgery trials
	-transplantation trials
	-transfusion trials
	-trials with cell therapy (when the cell preparation is not considered as an investigational medicinal product)
	-physical therapy trials
	-psychotherapy trials (without medicinal product)

**Diagnostic studies**	-diagnostic or imaging studies without medicinal product or medical device

**Clinical research on nutrition**	-nutritional studies
	-studies on nutritional supplements

**Other interventional clinical research**	-complementary and alternative medicine,
not using medicinal products nor medical devices	-collection of blood or tissue samples or other fluids
	-physiology studies
	-physiopathology studies
	-psychology studies.

**Epidemiology**	-interventional and non-interventional pharmacoepidemiology
	-interventional and non-interventional epidemiology
	-retrospective studies
	-registries of patients

The criteria to classify the different types of research are not uniform in Europe so that the appropriate rules can be applied in each case. The 2001/20/EC Directive defines two distinct categories of research: clinical trials (using an investigational medicinal product) and non-interventional trials (without medicinal product, without additional diagnostic or monitoring and where a medicinal product is used according to market authorisation). These definitions are interpreted differently from country to country leading some clinical research to be considered as clinical trial in one country and as non-interventional trial in another.

An intervention can be considered from a methodological point of view, that is the introduction of a procedure dictated by a clinical research protocol, e.g., allocation to an intervention arm by randomisation, while from a health-care point of view an intervention relates to how invasive or dangerous it is for the participants (e.g., in relation to collecting additional blood samples or performing additional diagnostic tests, or to the insufficiency of previous safety knowledge on the studied drug or procedure). The clinical definition of an intervention is independent of the study design. Unfortunately, the 'legal' definition of 'non-interventional', as described in the EU Directive 2001/20/EC for clinical trials with medicinal products appears to mix up both criteria, which make the interpretation difficult. In future regulatory definitions it would be desirable to incorporate all these aspects into a more meaningful concept. We also propose to separate therapeutic intervention compared to diagnostic intervention.

In addition to our concept of seven categories, we advocate protecting the enrolled participants using categories of perceived risk (understood as probability and severity of potential harm). Some member states (e.g., France) are already considering the risk-based approach. A related issue refers to what is meant by epidemiological studies or methods since it can be understood as synonymous to observational studies, i.e., non-interventional in the methodological sense. It would be better if future legal categories will define clinical study categories terms operationally rather than semantically.

### Survey

As the objective of the survey is to provide a comprehensive status of regulatory requirements the questions focused on the protocol submission to ethics committees and competent authorities, procedures for amendments, requirements for sponsor and insurance, and adverse event reporting applied to each clinical research categories. The main topics are summarised in the Appendix.

In addition a number of more specific questions were raised as well as suggestions for amendments to EU clinical research and its regulatory process.

The resulting survey served well its purpose, which will be described in future publications.

## Conclusion

Further work on the classification of clinical categories proposed here is necessary both to avoid unjustified overlapping of categories and to ensure that the definitions are as consistent as possible and correspond to existing scientific and legal thinking. We could also consider other existing proposals for classification. The UK Clinical Research Collaboration has developed the Health Research Classification System for the classification and analysis of all types of health research [8]. The system is two dimensional, classifying research according to the type of research taking place (research activity codes) and by area of health and disease (health categories). The eight main research activity codes are underpinning research, aetiology, prevention of disease and conditions, and promotion of well-being, detection screening and diagnosis, development of treatments and therapeutic interventions, evaluation of treatments and therapeutic interventions, management of diseases and conditions and health and social care services research. This listing shows that the research activity codes are less oriented to clinical trial structure but covers health care research as a whole.

ECRIN is currently cooperating with the European Medical Research Council (EMRC) on its forward looks on investigator-driven clinical trials. This document identified the adoption of common categories of clinical research as a high priority need, and has proposed to adopt the ECRIN categories [9,10].

In conclusion, the generation of an international survey pointed us to the important necessity to harmonise definitions and clinical trials categories in Europe to create the basis of a common understanding between international investigators and to simplify the preparation of international study protocols. We believe that the list of clinical research categories we have used for the ECRIN survey could serve as a contribution to the, much needed, task of harmonisation and simplification of the regulatory requirements for clinical research within the EU even if further efforts are necessary as discussed. It can initiate discussion and help reach agreement on common definitions of clinical trials terms and clinical research categories, both being prerequisite to conduct efficiently international clinical trials.

The ECRIN group will remain actively involved in the field in order to increase its scope and specifically address also the needs of countries currently outside its network. Therefore, all documents developed by ECRIN will be available at the ECRIN website  to stimulate discussion and exchange of ideas.

## Competing interests

The authors declare that they have no competing interests.

## Authors' contributions

CK, CG and JDM drafted the survey and conducted the analysis of the results. All the authors participated in the development of the survey and discussion of the results. All the authors have read and corrected draft versions and approved the final version.

## Appendix

### ECRIN survey

#### QUESTIONS ASKED TO EACH CATEGORY AND SUBCATEGORY OF CLINICAL RESEARCH AS DEFINED IN TABLE 1

Is a submission to an ethics committee required? (specify the name of the committee and who is responsible for the submission)

Is a submission to competent authority required? (specify the name of the competent authority and who is responsible for the submission)

Is there a specific procedure for substantial amendments?

Is there a requirement for a sponsor in this type of trial?

Is co-sponsorship allowed?

Is insurance required? (specify who is covered: sponsor, investigator, patients)

Adverse event (AE) reporting (Serious adverse events/Non serious adverse events)

Specify which adverse events have to be reported by the sponsor (or, if no sponsor, by the investigator) when and to whom? Is a safety report requested

#### ADDITIONAL QUESTIONS

➢ Is there a definition for interventional vs. non-interventional (or observational) clinical research?

➢ Are studies on usual care/quality studies/clinical audits considered as a specific category?

➢ Is there a definition for non-commercial trials?

➢ Is there a definition for a non-commercial sponsor?

➢ What is the definition of investigational medicinal products (IMP) in your country?

➢ Are there specific requirements for IMP labelling in trials on medicinal products?

➢ Are there specific requirements for IMP labelling in non-commercial trials?

➢ In non-commercial trials, is there a waiver for the sponsor to purchase the IMP?

➢ Are there specific requirements regarding compassionate studies/use?

➢ Are there any additional requirements for studies on biopharmaceuticals (proteins, monoclonals, DNA.)?

➢ Are there any additional requirements for studies on biotherapy (gene-cell-tissue)?

➢ Are there specific requirements for studies using adult stem cells?

➢ Are there specific requirements for studies using embryonic stem cells?

➢ Are there specific requirements for the *in vivo *use of nanoparticles (for diagnostic or treatment)?

➢ Are there specific requirements for studies using animal derived products?

➢ Are there requirements for specific populations? (healthy volunteers/Vulnerable populations)

➢ Are there specific requirements for emergency condition or critically ill patients?

➢ Is there a waiver of informed consent under emergency condition or critically ill patients?

➢ Are minority/ethnicity/gender taken into account in the national legislation?

➢ Is there a national volunteer's file for participants in clinical research?

➢ Are there compensation fees for volunteers/patients participating in clinical research?

➢ Are there specific strategies for monitoring clinical trials?

➢ Are there regulatory requirements regarding data management in clinical trials?

➢ Are there specific requirements regarding personal data protection in clinical research?

➢ Are there specific requirements regarding blood/tissue samples (circulation and storage)?

➢ Are there specific requirements regarding studies on biomarkers/surrogate markers (definition or validation of biomarkers)?

➢ Are there specific requirements regarding genetic or genotype/phenotype studies?

➢ Is there a national plan in your country on where to register clinical trials (a register where trial information can be made publicly available before inclusion of the first participant)?

➢ Is there a national plan on where to register anonymised data from the trial once it has been conducted and analysed?

➢ Is there a national plan on where to register publications deriving from the clinical trial?

➢ Is there an obligation to inform the patients on the outcome of the clinical trial?

➢ Does the legislative system in your country cover any biomedical research? or is it focusing on clinical research on health products?

#### COMMENTS

➢ Specify the five top priority topics to improve clinical research and provide suggestions for improvement

➢ Specify the five top priority topics to improve European competent authority working practice and provide suggestions for improvement

➢ What would be your expectations regarding future EU regulation on clinical research?
